# A Coherent Parameter Estimation Method for Distributed Coherent Jamming Systems

**DOI:** 10.3390/s26051655

**Published:** 2026-03-05

**Authors:** Liang Qi, Jianjiang Zhou

**Affiliations:** 1 School of Electronic Information Engineering, Nanjing University of Aeronautics and Astronautics, Nanjing 210016, China; 2 No. 723 Institute of China State Shipbuilding Corporation Limited, Yangzhou 225101, China; zjjee@nuaa.edu.cn

**Keywords:** distributed coherent jamming system (DCJS), coherent parameters estimation, generalized cross-correlation, synthesis efficiency

## Abstract

**Highlights:**

**What are the main findings?**

- A coherent parameter estimation method based on frequency-domain feature matching is proposed. A weighting method based on frequency-domain feature matching can effectively preserve the intra-pulse features of the signal, thereby improving the accuracy of coherent parameter estimation.
- The simulation results demonstrate that, compared with the GCC algorithm, the proposed method improves the time delay estimation accuracy by 27.0% and the phase difference estimation accuracy by 8.3%.

**What are the implications of the main findings?**

- The proposed method provides a robust estimation approach for coherent parameter estimation in distributed coherent jamming systems, and can significantly improve the estimation accuracy of time delay and phase difference.
- The proposed method exhibits a strong universality. With adaptive modifications, it can be extended to other signal processing fields where time delay and phase difference estimation are required.

**Abstract:**

Regarding the problem of the accurate estimation of coherent parameters for the distributed coherent jamming system (DCJS) in active radar applications, this paper first establishes a transmit–receive signal model of the DCJS in the presence of coherent parameter estimation errors. Then, it analyzes and verifies that the generalized cross-correlation function weighting method causes a decrease in the estimation accuracy of coherent parameters due to whitening processing, which in turn impairs the synthesis efficiency of the DCJS. Finally, a coherent parameter estimation method based on frequency-domain feature matching is proposed. The weighting method based on frequency-domain feature matching can effectively preserve the intra-pulse features of signals, thereby improving the estimation accuracy of coherent parameters. The simulation results show that, compared with the existing algorithms, the proposed method improves the time delay estimation accuracy by 27.0% and the phase difference estimation accuracy by 8.3%.

## 1. Introduction

Distributed coherent jamming is a crucial research direction in the field of electronic warfare, and the accurate estimation of coherent parameters is a prerequisite and core for achieving its excellent performance. Based on the distributed coherent synthesis technology [1], the distributed coherent jamming system (DCJS) enables each distributed aperture to conduct an accurate estimation of parameters such as the time delay and phase difference for received radar signals. According to the parameter estimation results, the transmission time and phase of jamming emission signals are precisely controlled to ensure that all jamming emission signals can arrive at the active radar target simultaneously and in-phase and achieve coherent synthesis. The primary objective of the DCJS is to realize the multiplication of jamming power for each distributed aperture (the jamming power can be increased by a factor of N^2^ when N jammers with the same effective radiated power are synthesized via distributed coherent technology), which can effectively address the bottleneck that the effective radiated power of a single jammer is difficult to further improve due to restrictions from the installation space, weight, power supply and other factors.

DCJS and distributed coherent aperture radar (DCAR) share many similarities, with the core issue being the estimation of coherent parameters (CPs) [2]. For DCAR to achieve fully coherent operation, the system first transmits orthogonal signals to accurately estimate time delay and phase difference. Based on the CP estimation results, the system performs time and phase compensation on the received signals to achieve receive coherence. Then, the system transmits the same waveform and adjusts the transmission time and phase using the obtained CP estimates to achieve transmit coherence, and again uses the received signals to update the CPs, performing time and phase compensation on the received signals to achieve full coherence [3,4,5,6,7].

However, the estimation of coherent parameters for the DCJS is entirely different from that for distributed coherent radar, and few open studies have reported research on the coherent parameter estimation of DCJS. By utilizing the spatiotemporal two-dimensional synchronous focusing characteristic of time-reversed electromagnetic waves, many researchers [8,9,10,11,12] have systematically proposed a spatial power synthesis method based on sparse arrays, and have carried out simulation analyses on the synthesis efficiency for single-frequency continuous wave signals, pulse signals and linear frequency-modulated signals. Meanwhile, they have proven that the spatial power synthesis method based on time reversal is also applicable to moving platforms. Some researchers [13,14,15] established a signal model for the spatial power combining of distributed jamming equipment based on the method of precise control of time and phase parameters, analyzed the impacts of time errors and phase errors on distributed spatial power combining, and provided a theoretical basis for the spatial power combining of distributed jamming equipment.

Cross-correlation-based methods can achieve an estimation accuracy approaching the Cramér–Rao bound under high signal-to-noise ratio (SNR) conditions, whereas their performance degrades drastically in high-noise environments. To mitigate the impact of Gaussian noise on time delay estimation results, Xing and Wang [16] proposed a method combining singular spectrum decomposition with an improved generalized cross-correlation algorithm. Experimental verification via multiple simulations under a −5 dB Gaussian noise environment demonstrated that, compared with the second-order correlation method, the absolute value of the main peak-to-sidelobe ratio is increased by more than 0.6756 dB. However, singular value decomposition involves extensive matrix operations, which makes it difficult to meet the real-time requirements of electronic warfare systems. To address the issue of noise interference on the phase spectrum, Guan Yu et al. [17] proposed an improved generalized cross-correlation method based on frequency-domain threshold processing. By setting a threshold on the amplitude spectrum prior to the weighting function operation, only the components of the cross-power spectrum function with an amplitude exceeding the threshold are retained in the frequency domain, thereby reducing the impact of noise on the phase spectrum. Nevertheless, this method is only applicable to narrowband signals, and its performance in coherent parameter estimation for wideband signals is not analyzed in the relevant literature.

Aiming at the problems of poor noise adaptability, low real-time performance and unsatisfactory estimation accuracy for practical applications existing in the current coherent parameter estimation methods, this paper proposes a feasible improved method for coherent parameter estimation on the basis of research into the generalized cross-correlation method and verifies the effectiveness and feasibility of the proposed improved method via simulation experiments.

## 2. Materials and Methods

This section elaborates on the proposed coherent parameter estimation method for improving the frequency-domain weighting function based on frequency-domain feature matching. Section 2.1 describes the transmit–receive signal model of the distributed coherent jamming system (DCJS) in the presence of coherent parameter estimation errors and derives the Cramér–Rao bound for coherent parameter estimation. Section 2.2 describes the conventional coherent parameter estimation method based on the generalized cross-correlation function, presents the method for improving the frequency-domain weighting function via frequency-domain feature matching, and performs coherent parameter estimation using the improved frequency-domain weighting function.

### 2.1. Signal Model of the Distributed Coherent Jamming System

**Figure 1:**
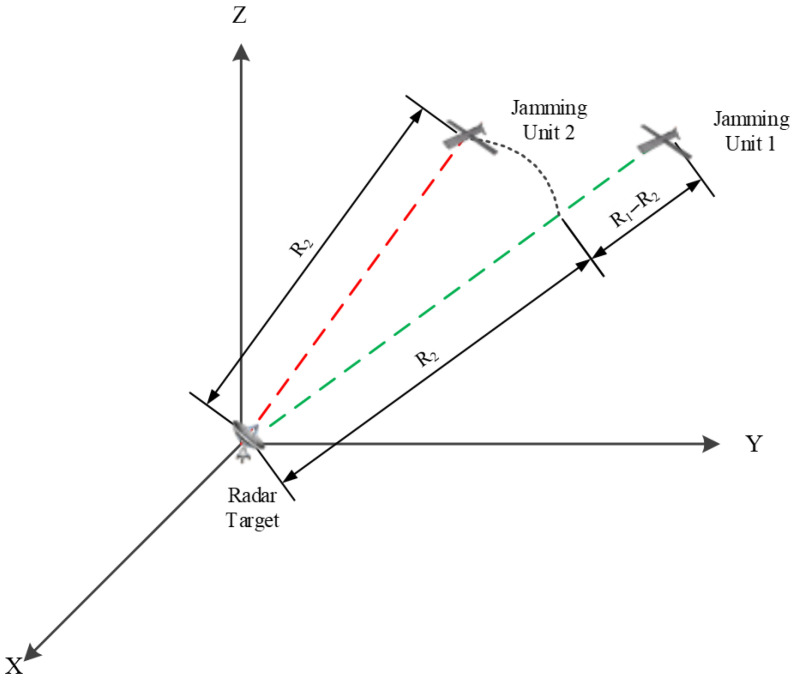
Schematic diagram of the spatial positions of jamming units and the radar target.

The schematic diagram of the jamming operation against radar implemented by a DCJS composed of two jamming units is illustrated in Figure 1. At time T_0_, the radial distance between jamming unit 1 and the radar target is $R_{1}$; jamming unit 1 moves at a constant speed $v_{1}$, with a radial velocity relative to the radar target of $v_{r1}$. The radial distance between jamming unit 2 and the radar target is $R_{2}$; jamming unit 2 moves at a constant speed $v_{2}$, with a radial velocity relative to the radar target of $v_{r2}$. The initial distance difference between the two jamming units and the radar target is $∆R=R_{1}-R_{2}$.

The signal transmitted by the radar target is expressed as follows:

(1) $$s_{tr}\left(t\right)=Au\left(t\right)e^{j2\pi f_{0}t}$$

where A denotes the signal amplitude, $A=\sqrt{P_{tr}G_{tr}Z_{tr}}$; $P_{tr}$ represents the radar transmitter power; $G_{tr}$ is the radar antenna gain; $Z_{tr}$ stands for the radar transmitting antenna impedance; $u\left(t\right)=rect\left(t/T_{p}\right)e^{j\pi kt^{2}}$ is the baseband signal; $T_{p}$ denotes the signal pulse width; k represents the signal frequency modulation slope, $k=B/T_{p}$; B is the signal bandwidth; $f_{0}$ stands for the signal carrier frequency; t is time; and tr stands for the transmitter.

The signal transmitted by the radar target propagates through space and reaches the two jamming units respectively. Due to the independent clocks and local oscillators of the two jamming units, there exist time and phase synchronization errors.

Assuming that the time synchronization error of jamming unit 2 relative to jamming unit 1 is ∆τ and the phase synchronization error is ∆φ, the signals received by the two jamming units are, respectively,

(2) $$x_{r1}\left(t\right)=\alpha_{r1}s_{tr}\left(t-T_{1}\right)=\alpha_{r1}Au\left(t-T_{1}\right)e^{j2\pi f_{0}\left(t-T_{1}\right)}$$

(3) $$x_{r2}\left(t\right)=\alpha_{r2}s_{tr}\left(t-T_{2}-∆\tau \right)e^{-j∆\varphi }=\alpha_{r2}Au\left(t-T_{2}-∆\tau \right)e^{j2\pi f_{0}\left(t-T_{2}\right)-j∆\varphi }$$

where $T_{1}=R_{1}/\left(c+v_{r1}\right)$ and $T_{2}=R_{2}/\left(c+v_{r2}\right)$, with c denoting the propagation speed of electromagnetic waves; $\alpha_{1}$ represents the transmission loss corresponding to the propagation path of jamming unit 1, where $\alpha_{r1}=\frac{1}{4\pi T_{1}f_{0}}\sqrt{G_{Jr1}\frac{Z_{r1}}{Z_{t}}}$, $Z_{r1}$ is the receiving antenna impedance of jamming unit 1 and $G_{Jr1}$ is the receiving antenna gain of jamming unit 1; and $\alpha_{r2}$ stands for the transmission loss corresponding to the propagation path of jamming unit 2, where $\alpha_{r2}=\frac{1}{4\pi T_{2}f_{0}}\sqrt{G_{Jr2}\frac{Z_{r2}}{Z_{t}}}$, $Z_{r2}$ is the receiving antenna impedance of jamming unit 2, and $G_{Jr2}$ is the receiving antenna gain of jamming unit 2.

Define the CPs ∆T and ∆Φ, where ∆T denotes the time delay between the radar signals received by the two jamming units, and ∆Φ represents the phase difference between the radar signals received by the two jamming units. Thus, we have

(4) $$∆T=T_{1}-T_{2}-∆\tau$$

(5) $$∆\Phi =\Phi_{1}-\Phi_{2}-∆\varphi$$

where $\Phi_{1}=2\pi f_{0}T_{1}$, $\Phi_{2}=2\pi f_{0}T_{2}$.

To ensure that the distributed jamming system operates coherently, the jamming signals transmitted by the two jamming units must arrive simultaneously and in-phase at the phase center of the radar antenna. Clearly, the two jamming units need to estimate the time delay and phase difference in the transmitted signals based on the received radar signals.

Define the unknown parameter vector as follows:

(6) $$\Lambda ={\left[∆T,∆\Phi \right]}^{T}$$

Assume that the estimated value of the unknown parameter vector is $\hat{\Lambda }$; it holds that

(7) $$\hat{\Lambda }={\left[∆\hat{T},∆\hat{\Phi }\right]}^{T}$$

where $∆\hat{T}$ and $∆\hat{\Phi }$ are the estimated values of ∆T and ∆Φ, respectively; $∆\hat{T}=∆T-{∆T}_{\varepsilon }$; $∆\hat{\Phi }=∆\Phi -{∆\Phi }_{\varepsilon }$; and ${∆T}_{\varepsilon }$ and ${∆\Phi }_{\varepsilon }$ represent the estimation errors of time delay and phase difference, respectively.

Based on the above definition of CPs, the time and phase parameters to be estimated are relative values rather than absolute values. During jamming transmission, it is only necessary to take one jamming unit as a reference and ensure the relative time and phase relationships between other jamming units and the reference unit. Let the time compensation amount be ${∆T}_{c}$ and the phase compensation amount be $∆\Phi_{c}$. Then, the transmitted signals of jamming units 1 and 2 are, respectively,

(8) $$x_{t1}\left(t\right)=\eta_{1}x_{r1}\left(t\right)$$

(9) $$x_{t2}\left(t\right)=\eta_{2}x_{r2}\left(t-∆T_{c}\right)e^{j2\pi f_{0}t-j∆\Phi_{c}}$$

where $\eta_{1}$ and $\eta_{2}$ denote the amplitude modulation coefficients of jamming units 1 and 2, respectively. By substituting the expressions in Equations (3) and (4) into Equations (8) and (9), respectively, the modified expressions can be obtained:

(10) $$x_{t1}\left(t\right)=\eta_{1}\alpha_{r1}Au\left(t-T_{1}\right)e^{j2\pi f_{0}\left(t-T_{1}\right)}$$

(11) $$x_{t2}\left(t\right)=\eta_{2}\alpha_{r2}Au\left(t-T_{2}-∆\tau -∆T_{c}\right)e^{j2\pi f_{0}\left(t-T_{2}\right)-j∆\varphi -j∆\Phi_{c}}$$

Assume that the jamming response times of the two jamming units are identical, denoted as $T_{re}$. Within the time $T_{re}$, the change in radial distance between jamming unit 1 and the radar is $cT_{1}-v_{r1}T_{re}$, with the corresponding propagation delay variation being $T_{1}-v_{r1}T_{re}/c$; the change in radial distance between jamming unit 2 and the radar is $cT_{2}-v_{r2}T_{re}$, with the corresponding propagation delay variation being $T_{2}-v_{r2}T_{re}/c$. Thus, the combined signal of the transmitted signals from the two jamming units at the radar target is

(12) $$x_{tr}\left(t\right)=\alpha_{t1}x_{t1}\left(t-T_{1}+v_{r1}T_{re}/c\right)+\alpha_{t2}x_{t2}\left(t-T_{2}+v_{r2}T_{re}/c\right)$$

where $\alpha_{t1}$ denotes the propagation loss corresponding to the transmission path from jamming unit 1 to the radar, and $\alpha_{t2}$ represents the propagation loss corresponding to the transmission path from jamming unit 2 to the radar. Substituting $x_{t1}\left(t\right)$ and $x_{t2}\left(t\right)$ (the transmitted signals of the two jamming units) into the combined signal expression, respectively, and defining $A_{1}=\alpha_{t1}\eta_{1}\alpha_{r1}A$, $A_{2}=\alpha_{t2}\eta_{2}\alpha_{r2}A$, $∆t_{1}=2T_{1}-v_{r1}T_{re}/c$, and $∆t_{2}=2T_{2}-v_{r2}T_{re}/c$, we obtain

(13) $$x_{tr}\left(t\right)=A_{1}u\left(t-∆t_{1}\right)e^{j2\pi f_{0}\left(t-∆t_{1}\right)}+A_{2}u\left(t-∆t_{2}-∆\tau -∆T_{c}\right)e^{j2\pi f_{0}\left(t-∆t_{2}\right)-j∆\varphi -j∆\Phi_{c}}$$

To ensure the two jamming signals are combined simultaneously and in-phase, the following condition must be satisfied:

(14) $$∆t_{1}=∆t_{2}+∆\tau +∆T_{c}$$

(15) $$2\pi f_{0}∆t_{1}=\;2\pi f_{0}∆t_{2}+∆\varphi +∆\Phi_{c}$$

That is,

(16) $$∆T_{c}=∆t_{1}-∆t_{2}-∆\tau$$

(17) $$∆\Phi_{c}=2\pi f_{0}\left(∆t_{1}-∆t_{2}\right)-∆\varphi$$

Substituting ∆T and ∆Φ according to Equations (4) and (5), we obtain

(18) $$∆T_{c}=2∆T-\left(v_{r1}-v_{r2}\right)T_{re}/c+∆\tau$$

(19) $$∆\Phi_{c}=2∆\Phi -2\pi f_{0}\left(v_{r1}-v_{r2}\right)T_{re}/c+∆\varphi$$

Substituting Equations (16) and (17) into Equation (13), we get

(20) $$x_{tr\_ideal}\left(t\right)=\left(A_{1}+A_{2}\right)u\left(t-∆t_{1}\right)e^{j2\pi f_{0}\left(t-∆t_{1}\right)}$$

After receiving the signal, the radar performs matched filtering processing to obtain the output signal after pulse compression. The impulse response of the matched filter is the time-reversed conjugate of the transmitted baseband signal:

(21) $$h\left(t\right)=rect\left(t/T_{p}\right)e^{-j\pi kt^{2}}$$

Then, the signal resulting after passing this signal through the matched filter is

(22) $$s_{0\_ideal}\left(t\right)=\int_{-\infty }^{+\infty }x_{tr\_ideal}\left(\tau \right)h\left(t-\tau \right)d\tau$$

Substituting $x_{tr\_ideal}\left(t\right)$ and $h\left(t\right)$ into the equation, we obtain

(23) $$s_{0\_ideal}\left(t\right)=\int_{-\infty }^{+\infty }\left(A_{1}+A_{2}\right)rect\left(\left(\tau -∆t_{1}\right)/T_{p}\right)e^{j\pi k{\left(\tau -∆t_{1}\right)}^{2}}e^{j2\pi f_{0}\left(\tau -∆t_{1}\right)}rect\left(\left(t-\tau \right)/T_{p}\right)e^{-j\pi k{\left(t-\tau \right)}^{2}}d\tau$$

After simplification, we can obtain

(24) $$s_{0\_ideal}\left(t\right)=\left(A_{1}+A_{2}\right)T_{p}sinc\left[\pi B\left(t-∆t_{1}\right)\right]e^{-j2\pi f_{0}∆t_{1}}$$

After passing through the matched filter, the carrier frequency of the output signal is $f_{0}$, and the envelope modulation is a sinc function.

Due to the existence of an estimation error ${∆T}_{\varepsilon }$ between the time delay ∆T and its estimated value $∆\hat{T}$, as well as an estimation error ${∆\Phi }_{\varepsilon }$ between the phase difference ∆Φ and its estimated value $∆\hat{\Phi }$, the two jamming signals cannot be completely superimposed at the radar target, leading to energy loss. Substituting Equations (18) and (19) into Equation (13), respectively, we obtain

(25) $$x_{tr}\left(t\right)=A_{1}u\left(t-∆t_{1}\right)e^{j2\pi f_{0}\left(t-∆t_{1}\right)}+A_{2}u\left(t-∆t_{2}-2∆\tau -2∆T+\left(v_{r1}-v_{r2}\right)T_{re}/c\right)\;\;\cdot e^{j2\pi f_{0}\left(t-∆t_{2}\right)-j2∆\varphi -j\left(2∆\Phi -2\pi f_{0}\left(v_{r1}-v_{r2}\right)T_{re}/c\right)}$$

Substitute the estimated values $∆\hat{T}$ and $∆\hat{\Phi }$ into the above equation; the actual superimposed signal at the phase center of the radar antenna is obtained:

(26) $$x_{tr\_actual}\left(t\right)=A_{1}u\left(t-∆t_{1}\right)e^{j2\pi f_{0}\left(t-∆t_{1}\right)}+A_{2}u\left(t-∆t_{2}-2∆\tau -2∆\hat{T}+\left(v_{r1}-v_{r2}\right)T_{re}/c\right)$$

Substitute $∆\hat{T}=∆T-{∆T}_{\varepsilon }$, $∆\hat{\Phi }=∆\Phi -{∆\Phi }_{\varepsilon }$, $∆T=T_{1}-T_{2}-∆\tau$, and $∆\Phi =2\pi f_{0}\left(T_{1}-T_{2}\right)-∆\varphi$ into the above equation; after simplification, we obtain

(27) $$x_{tr\_actual}\left(t\right)=A_{1}u\left(t-∆t_{1}\right)e^{j2\pi f_{0}\left(t-∆t_{1}\right)}+A_{2}u\left(t-∆t_{1}+2{∆T}_{\varepsilon }\right)e^{j2\pi f_{0}\left(t-∆t_{1}\right)+j{2∆\Phi }_{\varepsilon }}$$

The output signal after passing through the matched filter is

(28) $$s_{0\_actual}\left(t\right)=\int_{-\infty }^{+\infty }x_{tr\_actual}\left(\tau \right)h\left(t-\tau \right)d\tau$$

Substituting $x_{tr\_actual}\left(t\right)$ and $h\left(t\right)$ into $s_{0\_actual}\left(t\right)$, we obtain

(29) $$s_{0\_actual}\left(t\right)=\int_{-\infty }^{+\infty }\left[A_{1}u\left(\tau -∆t_{1}\right)e^{j2\pi f_{0}\left(\tau -∆t_{1}\right)}+A_{2}u\left(\tau -∆t_{1}+2{∆T}_{\varepsilon }\right)e^{j2\pi f_{0}\left(\tau -∆t_{1}\right)+j{2∆\Phi }_{\varepsilon }}\right]\;\cdot rect\left(\left(t-\tau \right)/T_{p}\right)e^{-j\pi k{\left(t-\tau \right)}^{2}}d\tau$$

After simplification, we obtain

(30) $$s_{0\_actual}\left(t\right)=A_{1}T_{p}sinc\left[B\left(t-∆t_{1}\right)\right]e^{-j2\pi f_{0}∆t_{1}}+A_{2}T_{p}sinc\left[B\left(t-∆t_{1}+2{∆T}_{\varepsilon }\right)\right]e^{-j2\pi f_{0}∆t_{1}+j{2∆\Phi }_{\varepsilon }}$$

When the time delay $2{∆T}_{\varepsilon }$ between the two jamming signals is greater than the radar’s time delay resolution, the radar will distinguish the two jamming signals as two separate targets. In this case, discussing the coherent synthesis efficiency is meaningless. The time delay resolution of the radar is $\frac{1}{2B}$; therefore, the coherent synthesis efficiency should be discussed under the condition $2{∆T}_{\varepsilon }<\frac{1}{2B}$, i.e., ${∆T}_{\varepsilon }<\frac{1}{4B}$.

The coherent synthesis efficiency η is defined as the ratio of the actual combined output power to the ideal coherent combined power, which is used to evaluate the effectiveness of coherent beam combination:

(31) $$\eta =\frac{\int {\left|s_{0\_actual}\left(t\right)\right|}^{2}dt}{\int {\left|s_{0\_ideal}\left(t\right)\right|}^{2}dt}$$

Substitute $s_{0\_actual}\left(t\right)$ and $s_{0\_ideal}\left(t\right)$, respectively; after simplification, we obtain

(32) $$\eta =\frac{A_{1}^{2}+A_{2}^{2}+2A_{1}A_{2}cos\left({2∆\Phi }_{\varepsilon }\right)\cdot \frac{\int {\left|F\left(f\right)\right|}^{2}cos\left(4\pi f{∆T}_{\varepsilon }\right)df}{E}}{{\left(A_{1}+A_{2}\right)}^{2}}$$

where $F\left(f\right)$ is the Fourier transform of $sinc\left[B\left(t-∆t_{1}\right)\right]$, E is the energy of the signal $sinc\left[B\left(t-∆t_{1}\right)\right]$, and $E=\int {\left|sinc\left[B\left(t-∆t_{1}\right)\right]\right|}^{2}d\left[B\left(t-∆t_{1}\right)\right]$.

**Figure 2:**
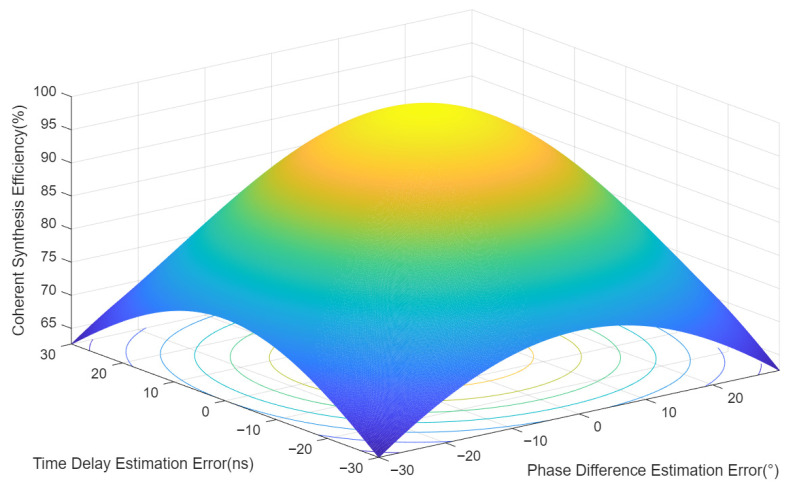
Curves of coherent synthesis efficiency versus time delay error and phase difference error.

**Figure 3:**
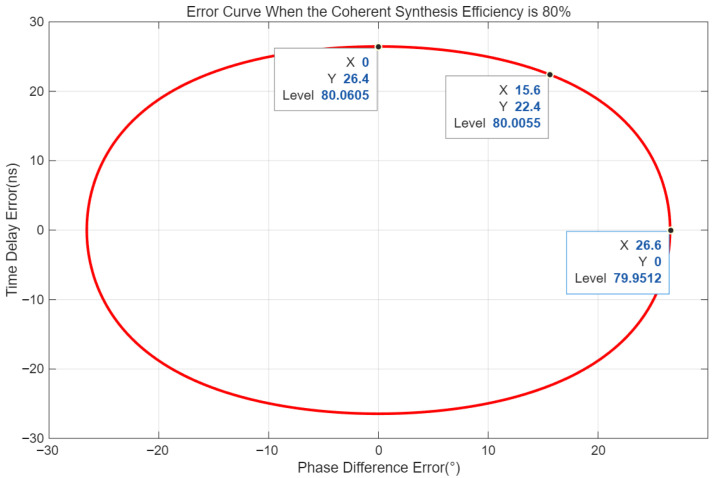
Error curves when coherent synthesis efficiency is 80%.

Obviously, when ${∆T}_{\varepsilon }\;=\;0$ and ${∆\Phi }_{\varepsilon }\;=\;0$, the coherent synthesis efficiency η = 1. Under different error conditions, a simulation analysis was performed on the coherent synthesis efficiency. The following assumptions are made: the distance between the two jamming units is 1000 m; the distance between jamming unit 1 and the radar target is 100 km; the distance between jamming unit 2 and the radar target is 100.1 km; the radar signal adopts linear frequency modulation (LFM), with a carrier frequency of 6 GHz, a bandwidth of 10 MHz, a pulse width of 10 μs, and a pulse repetition period (PRP) of 1 ms. The simulation results of the relationship between the time delay estimation error and the coherent synthesis efficiency are shown in Figure 2 and Figure 3.

Based on the above simulation results, the smaller the estimation errors of time delay and phase difference, the higher the coherent synthesis efficiency. Specifically, to achieve a coherent synthesis efficiency of 80% when the time delay estimation error is 0, the phase difference estimation error should be no more than 26.6°; when the phase difference estimation error is zero, the time delay estimation error should be no more than 26.4 ns; if the phase difference estimation error is 15.6°, the time delay estimation error should be no more than 22.4 ns. It can be seen that the estimation errors of time delay and phase difference directly affect the coherent synthesis efficiency. To achieve a coherent synthesis efficiency meeting the specified requirements, the estimation accuracy of time delay and phase difference must be controlled.

The Cramér–Rao low-bound (CRLB) for time delay and phase difference estimation can be readily derived as follows [18]:

(33) $${CRLB}_{∆T}=\frac{1}{SNR\cdot N}\cdot \frac{3}{\pi^{2}B^{2}}$$

(34) $${CRLB}_{∆\Phi }=\frac{1}{SNR\cdot N}$$

where SNR denotes the signal-to-noise ratio, N represents the number of nodes, and B stands for the signal bandwidth.

According to the above results, the CRLBs of time delay and phase difference have no correlation with the time synchronization accuracy ∆τ and phase synchronization accuracy ∆φ [19]. This seems contradictory to intuitive understanding because generally the worse the synchronization accuracy, the lower the estimation accuracy of time delay and phase difference will inevitably be. In fact, the time synchronization accuracy ‘∆τ’ and phase synchronization accuracy ‘∆φ’ between jamming units were taken into account in the above analysis. However, when solving the Fisher Information Matrix (FIM), $T_{2}+∆\tau$ and $\Phi_{2}+∆\varphi$ were analyzed as an integrated whole, thus avoiding the impact of ∆τ and ∆φ. Actually, the estimated parameters in the analysis are the estimates of the true time delay value $T_{1}-T_{2}-∆\tau$ and the true phase difference value $\Phi_{1}-\Phi_{2}-∆\varphi$. Specifically, the time delay estimation includes both the time delay caused by path difference and the time delay caused by clock asynchrony, while the phase difference estimation includes both the phase difference caused by path difference and the phase difference caused by clock asynchrony [20,21,22,23]. Therefore, the obtained CRLBs of time delay and phase difference are independent of the synchronization accuracy. In practice, however, synchronization errors are inevitably present in distributed coherent jamming systems. When adopting various coherent parameter estimation algorithms, the impact of synchronization accuracy on coherent parameter estimation must be considered [24,25].

### 2.2. Improvements to Coherent Parameter Estimation Method

On the basis of the traditional cross-correlation (CC) algorithm, the generalized cross-correlation (GCC) algorithm introduces a frequency-domain weighting process to suppress noise interference and enhance the effective components of signals. Its core idea is to perform frequency-domain pre-filtering on the two received signals, and then extract the time delay estimation value and phase difference estimation value through cross-correlation operation.

According to the Wiener–Khinchin theorem, the Fourier transform of the CC function is the cross-power spectrum of two signals; thus, we have

(35) $$G_{x_{1}x_{2}}\left(f\right)=\int_{-\infty }^{+\infty }R_{x_{1}x_{2}}\left(\tau \right)e^{-2\pi f\tau }d\tau$$

where $G_{x1x2}\left(f\right)$ is the cross-power spectral function of $x_{1}\left(t\right)$ and $x_{2}\left(t\right)$. Denoting $X_{1}\left(f\right)$ and $X_{2}\left(f\right)$ as the Fourier transforms of $x_{1}\left(t\right)$ and $x_{2}\left(t\right)$, respectively, we have $G_{x1x2}\left(f\right)=X_{1}\left(f\right)X_{2}^{*}\left(f\right)$. To suppress frequency-domain noise, a weighting function $\psi \left(f\right)$ is introduced to filter the cross-power spectral function $G_{x1x2}\left(f\right)$, resulting in the weighted cross-power spectral function [26]:

(36) $$G_{x_{1}x_{2}}^{g}\left(f\right)=G_{x_{1}x_{2}}\left(f\right)\psi \left(f\right)$$

According to the different construction methods of $\psi \left(f\right)$, commonly used weighting functions include the Phase Transform (PHAT), Smoothed Coherence Transform (SCOT), and Roth Weighting (ROTH) [27].

The weighting function of the PHAT method is the reciprocal of the modulus of the cross-power spectral function, i.e.,

(37) $$\psi_{PHAT}\left(f\right)=\frac{1}{\left|G_{x_{1}x_{2}}\left(f\right)\right|}$$

The PHAT method leverages the relationship between time delay and phase difference, i.e., φ=2πfτ. By directly normalizing the amplitude of the cross-power spectral function at all frequency points (a straightforward approach) and retaining only phase information, it can form a sharper cross-correlation function peak in the time domain. This is equivalent to performing whitening filtering on the signal. Under ideal conditions, the inverse Fourier transform of the cross-power spectral function weighted by PHAT is a Dirac delta function: the position corresponding to the peak is the time delay, and the phase corresponding to the peak is the phase difference—thereby enhancing the robustness of parameter extraction.

The PHAT method requires two FFT operations and one IFFT operation, with a computational complexity of $O\left(3NlogN+4N\right)$. However, the PHAT method completely discards amplitude information, which weakens the effective signal components and makes it sensitive to noise. Weighting amplifies phase errors in the noise frequency band, leading to an increase in the variance of time delay estimation.

The weighting function of the SCOT method is the reciprocal of the square root of the product of the auto-power spectra of the two signals, i.e.,

(38) $$\psi_{SCOT}\left(f\right)=\frac{1}{\sqrt{G_{x_{1}}\left(f\right)G_{x_{2}}\left(f\right)}}$$

where $G_{x_{1}}\left(f\right)={\left|X_{1}\left(f\right)\right|}^{2}$ and $G_{x_{2}}\left(f\right)={\left|X_{2}\left(f\right)\right|}^{2}$.

The SCOT method can eliminate frequency-domain distortion by normalizing the auto-power spectra of the two signals, allowing the CC function to focus more on time delay information. The weighted cross-power spectrum is equivalent to a coherence function, which reflects the linearity of the two signals at each frequency point. This enables the suppression of incoherent noise interference, making the SCOT method suitable for scenarios with frequency dispersion and capable of mitigating the impact of frequency-selective fading on time delay estimation. The computational complexity of the SCOT method is $O\left(3NlogN+6N\right)$. Similarly, the SCOT method suffers from noise sensitivity: at a low SNR, the auto-power spectrum estimation is contaminated by noise, resulting in degraded performance.

The ROTH method was first proposed in 1971 by Stanley A. Roth, a researcher from the Lincoln Laboratory at the Massachusetts Institute of Technology (MIT). Its weighting function is the reciprocal of the auto-power spectral function of the reference signal $x_{1}\left(t\right)$ [28], i.e.,

(39) $$\psi_{ROTH}\left(f\right)=\frac{1}{G_{x_{1}}\left(f\right)}$$

The core idea of the ROTH method is to suppress interference in noise-dominant frequency bands by normalizing the auto-power spectral function of the reference signal. Essentially, it assumes that noise mainly exists in the reference signal $x_{1}\left(t\right)$; through weighting, it suppresses the impact of low-noise frequency bands on cross-correlation. In the effective frequency bands of the signal (i.e., frequency bands with relatively high SNR), the weighted cross-power spectral function is approximately equal to $X_{2}\left(f\right)/X_{1}\left(f\right)$, which preserves the phase difference and time delay information of the two signals.

The computational complexity of the ROTH method is $O\left(3NlogN+3N\right)$. The ROTH method is suitable for scenarios where noise mainly exists in $x_{1}\left(t\right)$ while the noise in $x_{2}\left(t\right)$ is weak. It relies on the prior estimation of the noise power spectrum of the reference signal; otherwise, the weighting may fail.

**Figure 4:**
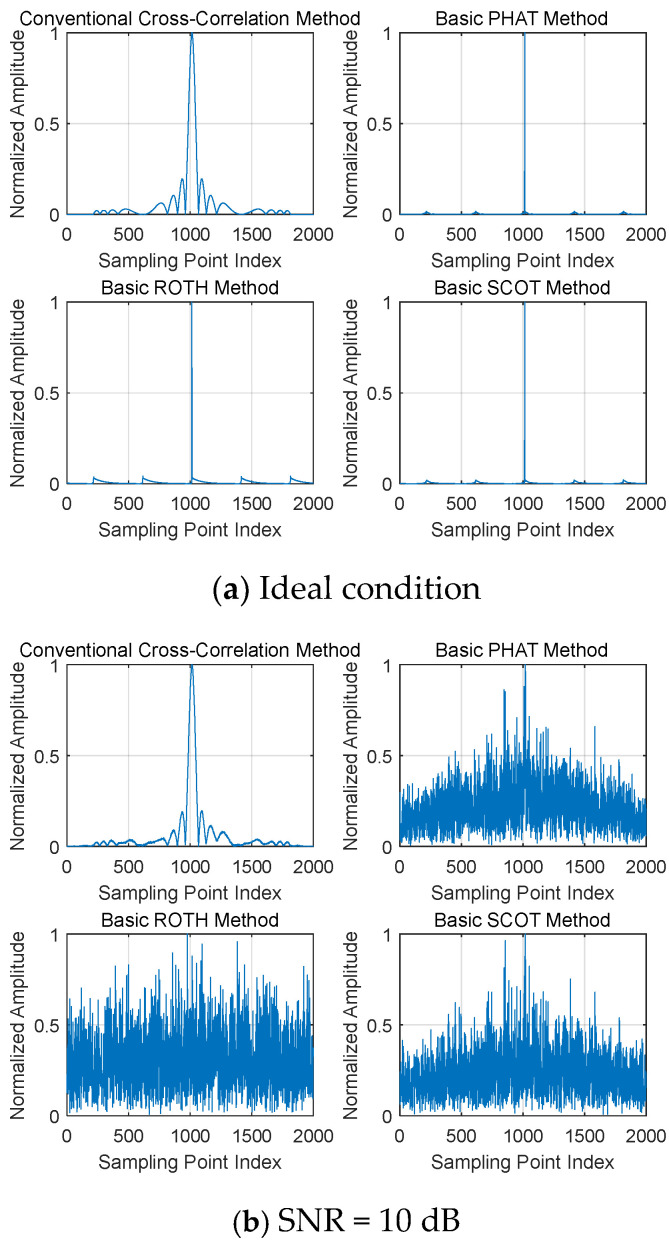
Comparison of CC functions between various weighting methods and the conventional CC method.

To intuitively demonstrate the effectiveness of each weighting method, Figure 4 presents the simulation results comparing the CC functions of each weighting method and the conventional CC method under the ideal no-noise condition and at an SNR of 10 dB.

As can be seen from Figure 4a, under ideal conditions, compared with the conventional cross-correlation function method, all weighting methods achieve the effect of peak sharpening to varying degrees. It can be observed from Figure 4b that, when the SNR is 10 dB, affected by noise, the noise floors of the CC functions of each weighting method rise to different extents. Among them, the ROTH method performs the worst, with its peak completely submerged by noise; the PHAT and SCOT methods perform slightly better. However, surprisingly, the conventional GCC method is almost unaffected by noise. Does this indicate that the generalized cross-correlation algorithm with frequency-domain weighting is ineffective? Actually, it does not.

As analyzed earlier, each weighting algorithm has its own distinct characteristics and applicable scenarios:

The ROTH method assumes that noise primarily exists in $x_{1}\left(t\right)$, which clearly does not align with the scenario of this study. In the scenario of this paper, it is assumed that there is additive white Gaussian noise (AWGN) with an independent and identical distribution (i.i.d.) in both $x_{1}\left(t\right)$ and $x_{2}\left(t\right)$; therefore, it is understandable that the ROTH method yields the worst performance.

The PHAT method performs whitening processing on the cross-power spectrum, which results in the loss of amplitude information at the target signal frequency. Consequently, the cross-correlation signal fails to obtain pulse compression gain, leading to a degraded performance when the SNR is not high.

The SCOT method normalizes the auto-power spectra of the two signals. In the scenario of this paper, the spectra of the two signals are consistent; thus, similar to the PHAT method, the CC signal loses pulse compression gain.

In contrast, the traditional CC algorithm can obtain full pulse compression gain. When the pulse width Tp is 10 μs and the bandwidth B is 10 MHz, an equivalent gain of 20 dB is achieved. Therefore, its performance is far superior to that of other frequency-domain weighted normalization algorithms.

In principle, the method of sharpening the CC function through frequency-domain weighting and normalization can certainly improve the robustness of time delay and phase difference estimation. However, the problem is that the above weighting algorithms discard the amplitude information in the frequency domain for both noise and signals through normalization, which will inevitably lead to corresponding losses. If we can only normalize the noise in the frequency domain while retaining the amplitude information of the useful frequency bands of the signal, it should be possible to improve the accuracy of parameter estimation.

For a linear frequency-modulated (LFM) signal, under ideal conditions, the amplitude of the cross-power spectrum is approximately rectangular, and the phase varies linearly with frequency. This is because the cross-power spectrum includes the phase difference term φ=2πfτ. Therefore, when the time delay τ is fixed, φ changes linearly with frequency, with a slope of 2πτ. Theoretically, the time delay value can be obtained by differentiating the phase of the cross-power spectrum, but this method has high requirements for the SNR [29].

Based on the cross-power spectral function of the two signals, the noise floor power is estimated, and a reference threshold is set according to the noise floor power estimation result. This threshold is used to protect the effective frequency bands of the signals: frequency-domain weighting processing is applied to frequency points whose power does not exceed the threshold, while frequency points whose power exceeds the threshold remain unchanged. By improving each weighting method, the following weighting functions are obtained:

(40) $$\psi_{PHAT\_IM}\left(f\right)=\left\{\begin{array}{c} \psi_{PHAT}\left(f\right),\;\left|G_{x1x2}\left(f\right)\right|\leq G_{n}\\ 1,else \end{array}\right.$$

(41) $$\psi_{SCOT\_IM}\left(f\right)=\left\{\begin{array}{c} \psi_{SCOT}\left(f\right),\;\left|G_{x1x2}\left(f\right)\right|\leq G_{n}\\ 1,else \end{array}\right.$$

(42) $$\psi_{ROTH\_IM}\left(f\right)=\left\{\begin{array}{c} \psi_{ROTH}\left(f\right),\left|G_{x1x2}\left(f\right)\right|\leq G_{n}\\ 1,else \end{array}\right.$$

where $G_{n}$ is the noise power threshold.

Through the above improved methods, the spectral characteristics of the normalized cross-power function are ensured to match those of the original cross-power spectrum, and the pulse compression gain of the cross-correlation function can be completely retained. Compared with the basic PHAT method, basic SCOT method, and basic ROTH method, the method of improving frequency-domain weighting based on frequency-domain feature matching only adds one comparison operation. The computational complexity of the improved method based on PHAT is $O\left(3NlogN+5N\right)$, the computational complexity of the improved method based on ROTH is $O\left(3NlogN+4N\right)$, and the computational complexity of the improved method based on SCOT is $O\left(3NlogN+7N\right)$.

## 3. Simulation-Based Verification

**Figure 5:**
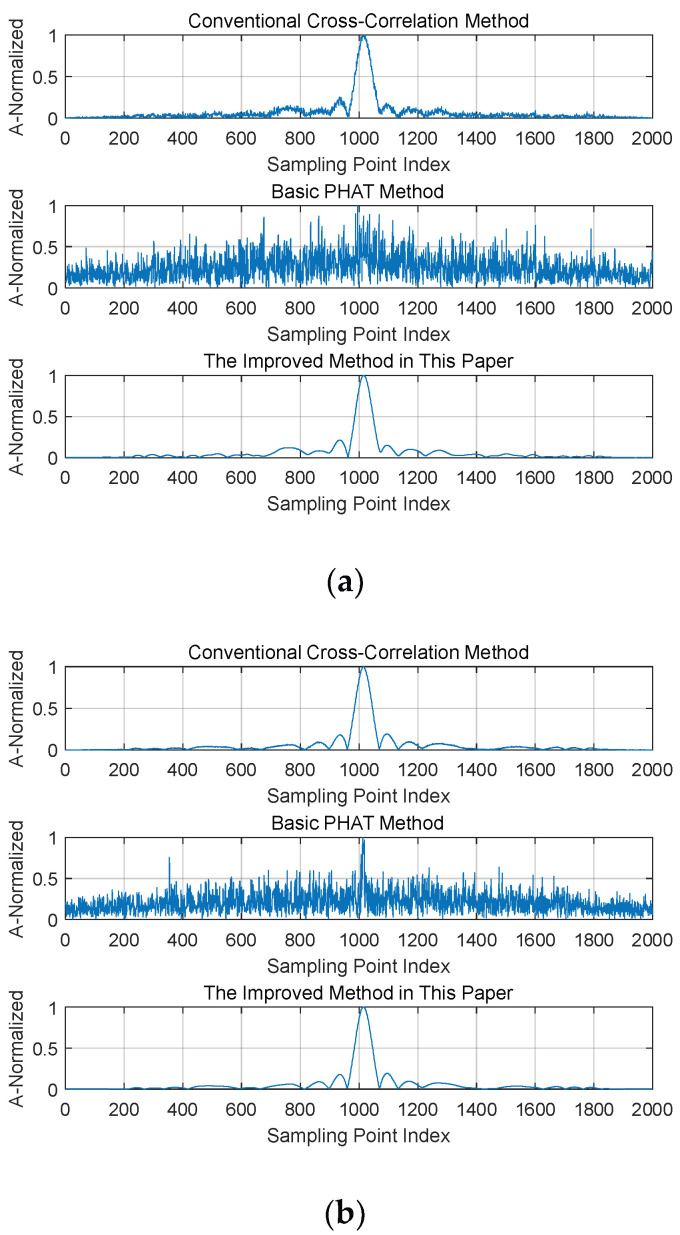
Comparison of cross-correlation function spectral peaks before and after improvement. (**a**) Comparison of PHAT Method Before and After Improvement (SNR = 0 dB). (**b**) Comparison of PHAT Method Before and After Improvement (SNR = 10 dB).

Based on the improved weighting functions, simulation and analysis were conducted on the CC functions and cross-power spectra of the PHAT-based improved method, ROTH-based improved method, and SCOT-based improved method, respectively. The signal parameters were set as follows: the pulse width Tp was 10 μs, the bandwidth B was 10 MHz, the sampling rate was 50B (i.e., 500 MHz), corresponding to a sampling interval of 2 ns, the data length N was 1000, the phase synchronization error ∆φ was 30°, the time synchronization error ∆τ was 5 ns, and the SNRs were 0 dB and 10 dB, respectively. The simulation results are shown in Figure 5; in each subfigure, the abscissa represents the number of sampling points, and the ordinate represents the normalized amplitude.

By comparing with the conventional cross-correlation function method, it can be observed that, in contrast to the original weighting function method, all improved weighting function methods can completely retain the pulse compression gain. Even under the low-SNR condition of 0 dB, the peaks of the cross-correlation function are still clearly visible and exhibit a better SNR than those obtained by the conventional cross-correlation function method. This indicates that the improved weighting function methods can filter out the influence of most noise while protecting the effective frequency band of the signal. Therefore, it can be inferred that the improved frequency-domain weighting algorithm can achieve a better coherent parameter estimation performance. In the following, the coherent parameter estimation performance of the improved weighting function method is simulated, and a comparative analysis is conducted with the basic generalized cross-correlation function method and the method proposed in [17].

**Figure 6:**
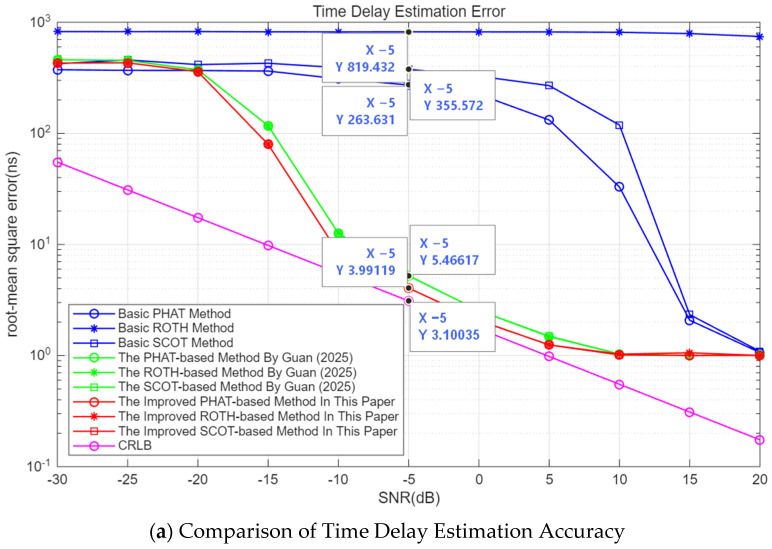

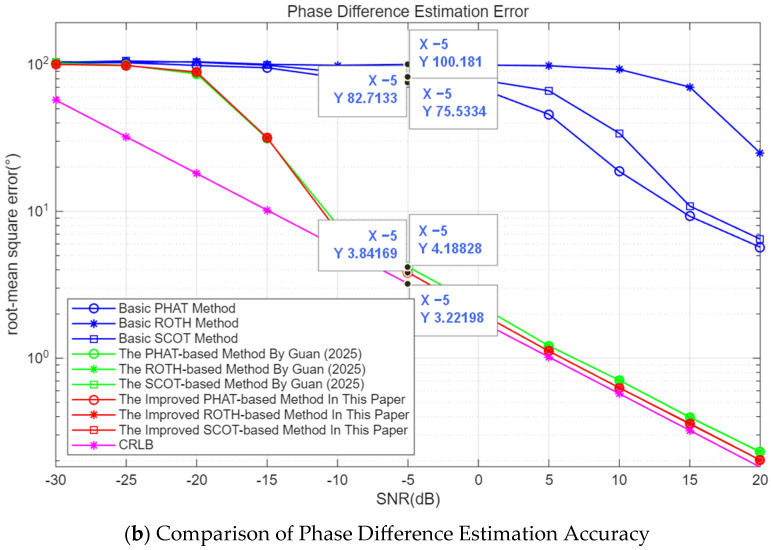
Comparison of estimation performance of the PHAT method before and after improvement (Method By Guan from [17]).

The simulation parameters remain unchanged, and 1000 Monte Carlo simulation experiments are conducted. The simulation results are shown in Figure 6. In Figure 6, the abscissa represents the SNR of the signal, and the ordinate represents either the time delay estimation accuracy (with the unit of ns) or the phase difference estimation accuracy (with the unit of °). The blue curve with circles denotes the basic PHAT method; the blue curve with stars denotes the basic ROTH method; the blue curve with squares denotes basic SCOT method; the green curves with circles represent the corresponding improved method from [17]; the red curves stand for the corresponding improved method proposed in this paper; and the magenta curve indicates the CRLB for CP estimation.

By comparing the blue curves in Figure 6a,b, it can be observed that the time delay estimation error and phase difference estimation error of the basic PHAT method and the basic SCOT method are lower than those of the basic ROTH method. This is consistent with the results obtained from the CC function, and the reason has been explained in the previous text, so it will not be repeated here.

By comparing the red curves, green curves, and blue curves in Figure 6a, it can be observed that the time delay estimation error of the PHAT-based improved method proposed in this paper is lower than that of the improved method from [17] and the basic PHAT method. When the SNR is −5 dB, the time delay estimation error of the PHAT-based improved method in this paper is 3.991 ns, which is much lower than that of the basic PHAT method (with a time delay estimation error of 263.6 ns). Compared with the improved method from [17] (with a time delay estimation error of 5.466 ns), the time delay estimation performance is improved by 27.0%, which verifies the effectiveness of the weighting function proposed in this paper for the PHAT method. Similar conclusions can be obtained for the PHAT-based and SCOT-based methods.

By comparing the red curves, green curves, and blue curves in Figure 6b, it can be seen that the phase difference estimation error of the PHAT-based improved method in this paper is slightly lower than that of the improved method from [17] and much lower than that of the basic PHAT method. When the SNR is −5 dB, the phase difference estimation error of the PHAT-based improved method in this paper is 3.842°, which is far lower than that of the basic PHAT method (with a phase difference estimation error of 75.53°). Compared with the improved method from [17] (with a phase difference estimation error of 4.188°), the phase difference estimation performance is improved by 8.3%. Similar conclusions can be obtained for the PHAT-based and SCOT-based methods.

As evident from Figure 6, for estimating time delay and phase difference, the estimation errors remain largely consistent across various SNRs, despite employing different weighting methods. This consistency arises because, in the scenario addressed by this paper, the received signals $x_{1}\left(t\right)$ and $x_{2}\left(t\right)$ from interference units share the same frequency spectrum, and the noise is independently and identically distributed (i.i.d.). Consequently, irrespective of the normalization technique applied, the denominator term of the weighting function remains the same when noise effects are disregarded, equating to the amplitude of the signal’s auto-power spectrum. Frequency-domain protection processing eliminates the impact of most noise frequency bands. Consequently, the resulting auto-correlation function not only maximally attenuates the noise but also preserves the pulse compression gain of the original signal. Hence, the enhanced frequency-domain weighting method proposed in this paper represents a more fundamental form of generalized correlation processing.

Ref. [17] combines the generalized cross-correlation algorithm with the least mean square time delay estimation (LMSTDE) method, forming an adaptively implemented generalized cross-correlation time delay estimation method, namely LMS-GCC. The computational complexity of the method in [17] is $O\left(N^{2}+KN\right)$, which is dominated by two key components: the generalized cross-correlation operation and the adaptive iteration of the LMS algorithm. For two received signal sequences, each of length N:

The direct time-domain GCC (adopted in LMS-GCC for adaptive implementation) has a time complexity of O(N^2^);

The LMS adaptive iteration requires K iterations (where K is the number of LMS iterations, typically K ≪ N), with each iteration contributing O(N) complexity (weighted summation, error calculation, and weight update). Thus, the total complexity of LMS iteration is O(KN).

Overall, the dominant term is the GCC operation, so the total computational complexity of the method in [17] is O(N^2^).

In contrast, our proposed method eliminates the frequency-domain GCC, resulting in a computational complexity of O(NlogN)). This represents a significant reduction in computational cost compared to the O(N^2^) complexity of the method in [17], especially when N is large (e.g., N > 1024).

Although the proposed method shows a modest gain over the known method, it achieves this with a significantly lower computational complexity and higher robustness under low SNR or complex environments. This trade-off is crucial for practical real-time applications, where computational efficiency and stability are often prioritized over marginal performance gains.

## 4. Conclusions

Addressing the core issue of coherent parameter estimation for distributed coherent jamming systems, this paper begins with the operational workflow of such systems and presents a corresponding signal model. Considering time synchronization errors and phase synchronization errors, the paper derives the CRLB for the estimation of coherent parameters (including time delay and phase difference), providing theoretical guidance for engineering implementation. Combined with practical engineering application scenarios, the theoretical feasibility of the distributed coherent jamming system is analyzed. Based on the implementation process of the system, it is pointed out that the primary factors affecting time synchronization accuracy and phase synchronization accuracy are the short-term frequency stability of the frequency source—this insight offers a theoretical basis for the engineering implementation of high-precision coherent parameter estimation in distributed systems.

Regarding the problem of coherent parameter estimation methods, the paper analyzes the cross-correlation function characteristics and amplitude-phase characteristics of cross-power spectra for four methods: the cross-correlation function method, PHAT method, ROTH method, and SCOT method. It identifies the limitations of these existing methods and proposes a time delay and phase difference estimation method based on frequency-domain threshold processing. This proposed method preserves the effective frequency band of the signal and performs noise whitening through frequency-domain thresholding, thereby fully preserving the pulse compression gain of the signal while suppressing noise.

The proposed method is applied to the PHAT, ROTH, and SCOT methods, respectively. Monte Carlo simulation results demonstrate that the coherent parameter estimation accuracy is significantly improved after integrating the proposed method: compared with the basic generalized cross-correlation method, the time delay estimation accuracy is improved by 27.0%, and the phase difference estimation accuracy is improved by 8.3%. Both theoretical analysis and simulation results confirm that the method proposed in this paper is a more fundamental generalized correlation algorithm.

In this study, the effects of different distances and angles on the estimation accuracy of time delay and phase difference are equivalently incorporated into the SNR parameter. In practical scenarios, the distances from different jamming nodes to the radar target cannot be exactly the same, and there are also differences in jamming response time and time synchronization accuracy among jamming nodes. All these factors will affect the estimation accuracy of time delay and phase difference. In future work, it is necessary to investigate the coherent parameter estimation accuracy under more scenarios, such as different jamming node configurations, different jamming response times, and different time synchronization accuracies.

## Data Availability

Data are contained within the article.
